# Dissimilar Fitness Associated with Resistance to Fluoroquinolones Influences Clonal Dynamics of Various Multiresistant Bacteria

**DOI:** 10.3389/fmicb.2016.01017

**Published:** 2016-07-07

**Authors:** Miklos Fuzi

**Affiliations:** Institute of Medical Microbiology, Semmelweis UniversityBudapest, Hungary

**Keywords:** fluoroquinolones, multiresistant, fitness, clone, incidence

## Abstract

Fitness cost associated with resistance to fluoroquinolones was recently shown to vary across clones of methicillin-resistant *Staphylococcus aureus* and extended-spectrum β-lactamase-producing *Klebsiella pneumoniae*. The resulting dissimilar fitness should have influenced the clonal dynamics and thereby the rates of resistance for these pathogens. Moreover, a similar mechanism was recently proposed for the emergence of the H30 and H30R lineages of ESBL-producing *E. coli* and the major international clone (ribotype 027) of *Clostridium difficile*. Furthermore, several additional international clones of various multiresistant bacteria are suspect to have been selected by an analogous process. An ability to develop favorable mutations in the gyrase and topoisomerase IV genes seems to be a prerequisite for pathogens to retain fitness while showing high-level resistance to fluoroquinolones. Since, the consumption of other “non-fluoroquinolone” groups of antibiotics have also contributed to the rise in resistance rates a more judicious use of antibiotics in general and of fluoroquinolones in particular could ameliorate the international resistance situation.

## Introduction

Though, clonal spread has always been a hallmark of many serious pathogens it is striking and remains enigmatic why the clonal spectra of several multiresistant bacteria have undergone a reduction at some point during the last three decades. We have witnessed a worldwide clonal shrink among others in methicillin-resistant *Staphylococcus aureus* (MRSA), extended-spectrum β-lactamase (ESBL)-producing *Klebsiella* *pneumoniae* and *Clostridium difficile*. The question arises what sort of driving force(s) could have reshaped the clonal landscape?

It is well-established that some clones of MRSA have for some time been on the advance replacing others and disseminating in novel geographic areas. Among the foraying MRSA clones ST22 (EMRSA-15) and CC5 proved particularly adept. In Europe where solid, up-to-date information on the clonal distribution of MRSA is available they are well-established to have become the dominant clones at the expense of multiple others (Grundmann et al., 2014). In addition, the ST22 clone has lately proved the most common sequence type in the healthcare setting in Australia (Coombs et al., 2014), and Singapore (Hsu et al., 2015). CC5 was reported the most widespread MRSA clone in the healthcare setting in Africa (Abdulgader et al., 2015) and it is dominant or on the rise in several Asian countries (Chen and Huang, 2014). CC5 has also remained the second most common clone in invasive infections in the United States though a novel antibiotic resistant variant of the CC8 clone (USA300) has recently emerged as the most frequent type of MRSA in blood samples (Tenover et al., 2011). In contrast various long prevalent MRSA clones, especially ST30 and ST239, have been losing ground to or have been replaced by CC5 and ST22 strains in hospitals worldwide (Velazquez-Meza et al., 2004; Ma et al., 2006; Amorim et al., 2007; Conceição et al., 2007; Aires-de-Sousa et al., 2008; Knight et al., 2012; Espadinha et al., 2013; Lim et al., 2013; Coombs et al., 2014; Hsu et al., 2015; Lawes et al., 2015). Furthermore, the ST228 (South-German) clone is also on the retreat. It has been reported replaced by ST22 isolates in both German and Italian facilities (Albrecht et al., 2011; Baldan et al., 2012). The surveillance conducted by the European Staphylococcal Reference Laboratory Working Group observed the overall decline of these latter clones between 2006 and 2011 (Grundmann et al., 2014). In addition, it reported a decrease in the incidence of the ST8 clone on the continent (Grundmann et al., 2014).

A reduction has also been observed in the clonal spectrum of ESBL-producing *K. pneumoniae*. A few STs of the pathogen mostly genetically related to each other have become internationally dominant during the last decade (Damjanova et al., 2008; Lee et al., 2011; Woodford et al., 2011; Rodrigues et al., 2014; Park et al., 2015). Interestingly, a marked reduction in the abundance of ESBL-producing *K. pneumoniae* STs was associated with a shift in the type of ESBL produced: while prior to the clonal reduction ESBLs of the SHV group prevailed strains of the novel major international STs produce primarily CTX-M-15 enzymes (Damjanova et al., 2008; Woodford et al., 2011). In addition a sole sequence type of *K. pneumoniae*, ST258, related to the major international clones, contributed significantly to the dissemination of carbapenem resistance worldwide (Woodford et al., 2011; Chen et al., 2014).

Moreover, a few major clones of *C. difficile* also attained international prominance (Smits, 2013; Tickler et al., 2014; Valiente et al., 2014; Freeman et al., 2015).

Strikingly all of these events took place at some point during the last three decades.

## Experimental Procedures

### Methicillin-Resistant *Staphylococcus aureus*

**FIGURE 1:**
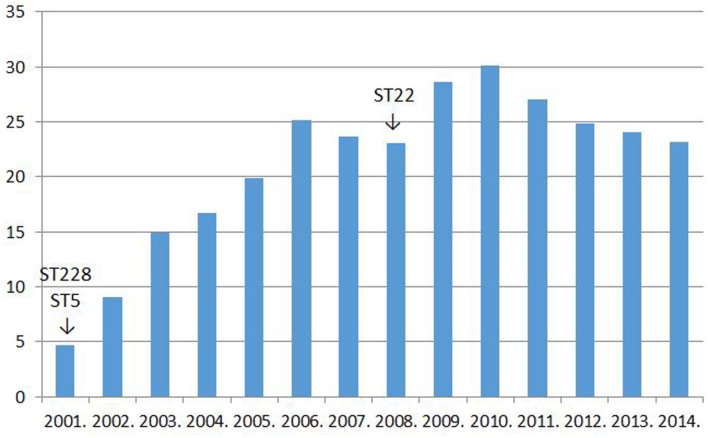
**The rate for MRSA among *Staphylococcus aureus* strains isolated from invasive infections in Hungary between 2001 and 2014% (http://ecdc.europa.eu/en/healthtopics/antimicrobial_resistance/database; see explanation in text)**.

Two clonal replacements have been observed in HA-MRSA in Hungary during the last 15 years. A dramatic shift took place about 15 years ago when strains of the New York–Japan (ST5) and South-German (ST228) clones almost completely supplanted isolates of the then resident Hungarian/Brazilian clone (ST239; Conceição et al., 2007). Moreover, we have been witnessing another MRSA clone replacement in Hungary during the last couple of years: the Western European clone ST22 (EMRSA-15) has been gradually expanding at the expense of mainly the South-German clone (ST228; Grundmann et al., 2014). Interestingly, both clonal shifts were associated with a transient increase in the rate for MRSA^1^. The incidence of MRSA from invasive infections in Hungary between 2001 and 2014 are shown in **Figure 1**.

Since, the observed clonal shifts in MRSA were supposed to have been promoted by varying fitness cost associated with resistance to some antibiotic the impact of fluoroquinolone resistance on the vitality of MRSA has been tested by us in a clonal affiliation (Horváth et al., 2012).

Propagation assays from various clones of MRSA showing similar MIC values for fluoroquinolones established that isolates from the New York–Japan (ST5) and South-German clones (ST228) retained significantly more fitness than strains from the Hungarian/Brazilian clone (ST239) which they replaced in Hungary (Horváth et al., 2012). In addition isolates from the EMRSA-15 clone (ST22) maintained more vitality than the subsequently supplanted South-German strains. Moreover, a fluoroquinolone resistant isolate from the ST30 CA-MRSA clone suffered much greater fitness cost than strains from any other clone tested. Furthermore, fluoroquinolone resistant strains from two additional CA-MRSA clones (ST8 and ST80) also displayed fitness inferior to that of the EMRSA-15 (ST22), New York–Japan (ST5) and South-German (ST228) strains, though, this difference was smaller than that observed with the ST30 and ST239 isolates (Horváth et al., 2012). These results should account not just for the clonal shifts observed in Hungary but also for the failure of the ST30, ST80, and ST8 CA-MRSA clones to disseminate in Hungarian hospitals.

The superior fitness shown by the newly invading MRSA clones should well-explain the increase in the rate for MRSA detected transiently subsequent to both clonal replacements (**Figure 1**).

**FIGURE 2:**
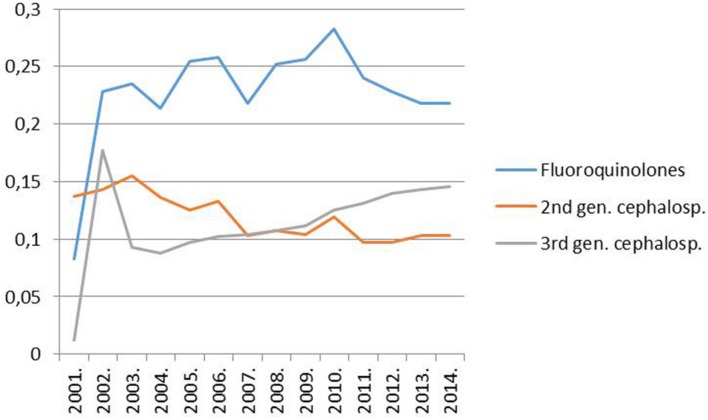
**Trend of the consumption of fluoroquinolones (ATC group J05), second-generation cephalosporins (ATC group J05) and third-generation cephalosporins (ATC group J05) in the hospital sector in Hungary from 1998 to 2014 (DDD per 1000 inhabitants and per year; http://ecdc.europa.eu/en/activities/surveillance/ESAC-Net/Pages/index.aspx)**.

However, it must have been a rise in the use of fluoroquinolones that could have allowed the widespread dissemination of the highly fluoroquinolone resistant major clones of MRSA in Hungary. Trends in the rate for MRSA indeed proved significantly associated with trends in the consumption of fluoroquinolone type antibiotics in the country (Pearson correlation, significance two-tailed: 0.03; **Figures 1** and **2**). The MRSA rates also displayed a significant association with the consumption of second generation cephalosporins though the relationship proved somewhat weaker (Pearson correlation, significance two-tailed: 0.05). The consumption of third generation cephalosprorins was unrelated to the rate for MRSA (Pearson correlation, significance two-tailed: 0.140)^1,^^2^.

Subsequently, Knight et al. (2012) investigating the clonal dynamics of MRSA in a British hospital published findings consistent with our results. In addition, Holden et al. (2013) showed that the development of resistance to fluoroquinolones played a pivotal role in the widespread dissemination of the ST22 clone. Moreover, Lawes et al. (2015) considered the use of fluoroquinolones significant in the clonal shifts of MRSA in Scotland and Hsu et al. (2015) also suggested that the use of fluoroquinolones could have contributed to a clonal rearrangement in MRSA in Singapore. Moreover, the results are propped up by substantial literature on the replacement of the ST30, ST239, ST228, and ST8 clones by ST22 and CC5 strains in the healthcare setting in various parts of the world referred to above.

Apart from demonstrating differences in the fluoroquinolone resistance-associated fitness cost between individual clones of MRSA our work also showed that some differences between isolates affiliated with the same STs also exist (Horváth et al., 2012). Thus, STs are having “sublineages” showing to some extent diverse fitness costs associated with resistance to fluoroquinolones. The recent dissemination of the originally CA-MRSA USA300 (CC8) clone in American hospitals should be related to the emergence of such a novel sublineage commanding better fitness relative to previous lineages when showing resistance to fluoroquinolones. It is probably no accident that Tenover et al. (2011) and Alam et al. (2015) both emphasized that in contrast to previous strains many of the recently tested USA300 isolates proved resistant to fluoroquinolones. Moreover, the emergence of a new sublineage within the USA300 clone should not come as a surprise since the clone ab ovo consisted of a genetically related but diverse group of strains (Tenover and Goering, 2009).

Though, a variety of pathogenicity factors and resistance markers to “non-fluoroquinolone” antibiotics should also affect the dissemination of MRSA the findings with fluoroquinolone resistance-associated fitness cost strongly suggest that it is the primary determinant of the epidemiology of MRSA in every area where fluoroquinolones remain in extensive use. There has to date been no report convincingly demonstrating that any other factor – with the obvious exception of the abandonment of the use of β-lactam antibiotics – could have exerted a more profound effect on the clonal dynamics of the pathogen in the healthcare setting.

Whole-genome sequencing to compare the genetic composition of various clones of MRSA and, thus, identify determinants influencing clonal dynamics have been performed by two groups. Hsu et al. (2015) reported on the displacement of the ST239 MRSA clone by the ST22 clone in Singapore. A thorough review of the genetic makeup of both clones interestingly showed that in contrast to the ST22 isolates many of the ST239 strains harbored genes of the “arginine catabolic mobile element” (ACME), nevertheless, they have been readily replaced by the ST22 isolates. No additional suspect pathogenicity factor could be demonstrated in either clones. However, authors hint that it could have been fitness cost associated with resistance to fluoroquinolones that was responsible for the success of the ST22 clone.

Moreover, Alam et al. (2015) genetically investigated the novel lineage (clade) of the USA300 clone that has recently been emerging in the USA. Though, whole-genome sequencing on many isolates have been performed they failed to identify any suspect pathogenicity factor that could have accounted for the novel clade’s dissemination. Nevertheless, they observed that, in contrast to strains of the old lineage, isolates of the emerging clade were all resistant to fluoroquinolones and harbored “classical” gyrase and topoisomerase mutations.

Though, the superior production of biofilm was reported to promote the dissemination of the ST22 (EMRSA-15) clone its general biofilm-producing capacity is in fact inferior compared with those of the ST228 and ST8 strains it readily replaces in the healthcare setting (Baldan et al., 2012).

Although, resistance markers to “non-fluoroquinolone” antibiotics should also impact the fitness of MRSA the preeminence of fluoroquinolone resistance-associated fitness cost is reflected in the observation that our CA-MRSA strains were usually more susceptible to various additional groups of antibiotics than the major clone HA-MRSA isolates, nevertheless, they suffered more fitness cost upon acquiring resistance to fluroquinolones than them. Interestingly, the CA-MRSA isolate suffering the most fitness cost subsequent to the induction of resistance to ciprofloxacin was the ST30 strain showing resistance exclusively to β-lactam antibiotics apart from fluoroquinolones (Horváth et al., 2012).

### ESBL-Producing *K. pneumoniae* and ESBL-Producing *E. coli*

A couple of years subsequent to the “epidemiological earthquake” observed with MRSA a major clonal shift took place with ESBL-producing *K. pneumoniae* in Hungary (Damjanova et al., 2006, 2008). Prior to 2003 ESBL-producing *K. pneumoniae* had been polyclonal in the country and the isolates produced SVH type enzymes (Damjanova et al., 2007). However, after 2004 we witnessed the emergence of three major STs of ESBL-producing *K. pneumoniae* two of which (ST11, ST15) were originally detected in France^3^. Interestingly, in contrast to previous isolates, all of the novel strains carried the CTX-M-15 ESBL gene (Damjanova et al., 2006, 2008). The new major clones disseminated exclusively in adult hospital ward where fluoroquinolones were in extensive use and not in the perinatal intensive care units where fluoroquinolones are not considered a drug of choice (Szilágyi et al., 2010). In the perinatal intensive care units the previous epidemiological situation prevailed: the isolates remained polyclonal and continued to produce SHV type enzymes (Szilágyi et al., 2010).

Similarly, to the MRSA situation the observed clonal change was associated with a rise in the rate for ESBL-producing *K. pneumoniae* in Hungary^4^ (Data not shown). This rise, however, in contrast to MRSA, was not expected to be significantly associated with the consumption of fluoroquinolones for two reasons.

(1) The new ESBL-producing *K. pneumoniae* clones “invaded Hungary” a couple of years subsequent to the increase in the consumption of fluoroquinolones and, thus, commenced to expand in 2004 after the advent of the widespread use of fluoroquinolones in the country (**Figure 2**; Damjanova et al., 2006, 2008^5^).
(2) The dissemination of the major ESBL-producing *K. pneumoniae* clones did not prove as exclusive as that of the major HA-MRSA clones in Hungary (Szilágyi et al., 2010) and rates for clones not selected by fluoroquinolones are not supposed to be governed by changes in the consumption of these agents.

Though, varying fitness cost associated with resistance to fluoroquinolones was obviously influencing the clonal dynamics of MRSA in the hospital setting we have demonstrated an even more pronounced difference between minor clone and major clone strains in ESBL-producing *K. pneumoniae*. Some of our minor clone *Klebsiella* strains, originally susceptible to fluoroquinolones, suffered a dramatic drop in fitness when resistance to ciprofloxacin was induced in them while others proved unable to assume high-level resistance to ciprofloxacin (Tóth et al., 2014). Interestingly, in contrast to major clone isolates which all carried three mutations in the gyrA and parC genes, the minor clone ESBL-producing *K. pneumoniae* strains either failed to develop any of the well-known gyrA and parC mutations or had fewer of them (Tóth et al., 2014).

Additional determinants of fluoroquinolone resistance were also investigated (Tóth et al., 2014). The qnrA, B, C, D, and S; qepA and oqxAB were not detected in any of our isolates. The aac(6′)-Ib-cr was demonstrated in all of the four major clone strains and in one of the minor clones isolates (the single strain carrying a CTX-M-15 plasmid). Moreover, an active efflux system was observed in three of the four minor clone ciprofloxacin resistant isolates but in none of the major clone strains (Tóth et al., 2014).

These results should account for the clonal dynamics of ESBL-producing *K. pneumoniae* in Hungary and could explain the widespread international dissemination of the CTX-M-15 enzyme. Nevertheless it remains to be established why major clone strains proved more adept in developing favorable mutations in the gyrA and parC genes than minor clone isolates. In addition the background of clonal affiliation of fitness cost elicited by the CTX-M-15 plasmid also needs to be elucidated.

Antibiotics other than fluoroquinolones have not been observed to appreciably impact the clonal dynamics of *K. pneumoniae*. Most of our minor clone *K. pneumoniae* isolates showed similar MIC values to aminoglycosides to those of major clone strains (Tóth et al., 2014). In addition, though three of the four minor clone isolates eliminated the SHV plasmids during the induction of resistance to ciprofloxacin which was associated with a dramatic drop in β-lactam resistance they suffered much greater fitness costs than the major clone strains carrying the CTX-M-15 plasmids and showing high level resistance to β-lactams (Tóth et al., 2014).

Apart from fitness cost associated with resistance to antibiotics virulence factors should also influence the clonal dynamics of ESBL-producing *K. pneumoniae*. Nevertheless data available in the literature strongly suggest that the role of virulence factors remains inferior relative to that of fitness cost associated with resistance to fluoroquinolones.

Lascols et al. (2013) compared the virulence arsenal of major and minor clone strains of ESBL-, and carbapenemase-producing strains of *K. pneumoniae* and observed that – in contrast to expectation – the minor clone isolates harbored somewhat more virulence factors. Andrade et al. (2014) hinted that the production of biofilm could have contributed to the success of the ST11 major clone of *K. pneumoniae* and Melegh et al. (2015) found that the major clone strains of *K. pneumoniae* were more likely to produce biofilm than minor clone isolates. However, Diago-Navarro et al. (2014) observed “heterogeneity” in the formation of biofilm in ST258 strains of *K. pneumoniae* – a close relative of the ST11 clone. Moreover, Kong et al. (2012) questioned the role of biofilm formation in the development of systemic infection with *K. pneumoniae*. Furthermore, Melegh et al. (2015) also observed that the major clone isolates of *K. pneumoniae* displayed significantly higher MIC values to ciprofloxacin than the minor clone strains!

A similar mechanism was reported by an American group for *E. coli* last year. The multiresistant ST131 clone of *E. coli* emerged as an international pathogen in 2008 (Nicolas-Chanoine et al., 2008) and contributed to the worldwide spread of the CTX-M-15 ESBL gene (Nicolas-Chanoine et al., 2014; Mathers et al., 2015). Johnson et al. (2015) recently demonstrated that the main multiresistant international lineages of the clone (H30, H30R) command a “fitness advantage” relative to isolates from other clones when showing high level of resistance to fluoroquinolones. This “fitness advantage” – similarly to major clone isolates of ESBL-producing *K. pneumoniae* – was associated with the quantity of favorable mutations in the *gyrA, parC*, and *parE* genes and a significantly weaker efflux activity relative to isolates from other lineages (Johnson et al., 2015). Furthermore a very recent revision of the topic based on the analysis of whole-genome sequences concluded that “strong selection pressure exerted by the widespread use of fluoroquinolones and extended-spectrum cephalosporins” “most likely” played a crucial role in the emergence of the H30 and H30R lineages (Stoesser et al., 2016).

### *Clostridium difficile* and Additional Pathogens

It is well-established that the acquisition of fluoroquinolone resistance is a novel characteristic in the major international ribotype 027 of *C. difficile* compared with earlier strains of the pathogen (He et al., 2013; Spigaglia, 2016). Moreover, Wasels et al. (2015) recently showed that resistance to fluoroquinolones in ribotype 027 strains is associated with a very modest fitness cost; a trait linked to the presence of a favorable mutation (Thre82Ile) in the gyrA gene. This result strongly argues for a mechanism similar to that observed with HA-MRSA and ESBL-producing *K. pneumoniae* since the same mutation has also been demonstrated in isolates of some additional international ribotypes genetically related or unrelated to ribotype 027 (Carman et al., 2009; Saxton et al., 2009; Spigaglia et al., 2010; Walkty et al., 2010; Lin et al., 2011; Solomon et al., 2011; Lee et al., 2014; Baldan et al., 2015; Kuwata et al., 2015).

The presence of the energetically favorable Thre82Ile gyrA mutation in many strains of the major international ribotypes of *C. difficile* should have promoted their dissemination in a fluoroquinolone affected area that may partly account for the relative diversity of the clonal spectrum of the pathogen (Bauer et al., 2011; Tickler et al., 2014; Freeman et al., 2015). This contrasts with the clonal landscape of ESBL-producing *K. pneumoniae* where the capacity of developing favorable gyrA and parC mutations seems to be the hallmark of just a few genetically related international STs (Tóth et al., 2014).

A well-documented clonal shift of *C. difficile* occurred in a Korean hospital reflecting the findings of Wasels et al. (2015). The earlier prevalent *C. difficile* ribotype 001 strains were replaced by isolates from the 014, 017, and 018 ribotypes within a couple of years (Lee et al., 2014). Interestingly, all of the novel ribotype strains carried the energetically favorable Thre82Ile gyrA mutation while the Korean ribotype 001 isolates harbored the Thre82Va gyrA mutation that has been shown by Wasels et al. (2015) to be associated with a significant fitness cost.

Moreover, the proposed “fluoroquinolone mechanism” is supported by the observation that the proportion of the ribotype 027 clone is significantly greater in adult ward than in pediatric units (McFarland et al., 2016).

Apart from favorable fitness various virulence factors produced by ribotype 027 strains have certainly contributed to the clone’s dissemination (Stabler et al., 2009; Valiente et al., 2014), although, differences between individual strains do exist (Carlson et al., 2013). Furthermore, strains of additional major ribotypes 001 and 106 have also been shown to command superior virulence relative to many other clones (Vohra and Poxton, 2011). Nevertheless Sarma et al. (2015) recently reported that a significant decrease in the consumption of fluoroquinolone type antibiotics resulted in the partial replacement of the ribotype 027, 001, and 106 strains – all reported to have been carrying the Thre82Ile gyrA mutation (Carman et al., 2009; Saxton et al., 2009; Cartman et al., 2010; Solomon et al., 2011) – by a variety of minor clones. Consequently, superior virulence could not prevent the demise of strains from any of these ribotypes once the selecting pressure of fluoroquinolone exposure ceased. Moreover, these findings are also in agreement with the national *C. difficile* statistics of the UK (Wilcox et al., 2012).

Furthermore, Wasels et al. (2015) demonstrated that the gyrB Asp426Asn and Asp426Val mutations confer an extra fitness on *C. difficile* irrespective of exposure to fluoroquinolones. These genetic alterations should also influence the clonal dynamics of the pathogen. However, to properly investigate their impact their prevalence should be investigated in a clonal affiliation.

In addition to the pathogens mentioned above the prospect of a “fluoroquinolone resistance-associated fitness mechanism” in the dissemination of a variety of multiresistant pathogens would be worth investigated. Among others the ST198 clone of *Salmonella* Kentucky (Le Hello et al., 2013) and the fluoroquinolone resistant clone of *Streptococcus pneumoniae* (Ben-David et al., 2014).

To our understanding there may be a single species of bacteria that seems to have efficiently adapted to resistance against fluoroquinolones and varying fitness cost has to date certainly failed to select a major international clone in it: *Campylobacter jejuni* (Luo et al., 2004; Zeitouni and Kempf, 2011). The excellent adaptation of *C. jejuni* is probably due to the hyperplasticity of its genome (Stahl and Stintzi, 2011).

In summary our results and those of others indicate that diverse fitness cost associated with resistance to fluoroquinolones influenced the evolution and extensive dissemination of the major international clones of a variety of important multiresistant pathogens.

## Practical Consequences and Discussion

Since the fitness of the major clones of multiresistant pathogens mentioned above proved superior to those of the previously prevalent minor clone isolates they may have disseminated more quickly and, thus, could have influenced the rates for the multiresistant pathogens in facilities/ward where fluoroquinolones remained in extensive use. This process, as indicated above, seemed obvious in Hungary with both MRSA and ESBL-producing *K. pneumoniae*.

However, if the extensive use of fluoroquinolones – and that of additional groups of antibiotics – contributed to a rise in the incidence of various multiresistant bacteria, then a more judicious consumption of antibiotics in general and of fluoroquinolones in particular should lower the rate of resistance for these pathogens. The available quantity and quality of information in this respect varies across species. The data on MRSA is the most abundant and seem to be the most appropriate for drawing inferences from. This is no accident since the selection of all major international clones of HA-MRSA have been influenced by fluoroquinolones. Though, most studies investigating a possible link between the consumption of fluoroquinolones and the rate for MRSA did not establish the clonal affiliation of the isolates at the facilities observed, basically all surveys necessarily investigated the prevalence of “fluoroquinolone-associated clone” isolates.

Most of the literature published on the influence of fluoroquinolone consumption on the rate for MRSA show a clear relationship: the more fluoroquinolones are used the higher the rate for MRSA will rise and vice versa. The association seemed so close that both the Society of Healthcare Epidemiology of America (SHEA) in its 2003 guideline (LeDell et al., 2003) and the British Department of Health in its 2011 guideline (Byrne and Wilcox, 2011) recommended a restriction in the use of fluoroquinolones as a control measure to curb the spread of MRSA. Moreover, an abundance of papers demonstrated a direct link between fluoroquinolone use and the incidence of MRSA between 1998 and 2015 (Hill et al., 1998; Crowcroft et al., 1999; Gruson et al., 2000; Harbarth et al., 2000; Graffunder and Venezia, 2002; Weber et al., 2003; Aubert et al., 2005; Nseir et al., 2005; Charbonneau et al., 2006; Cook et al., 2006, 2011; LeBlanc et al., 2006; Madaras-Kelly et al., 2006; Muller et al., 2006; Rogues et al., 2007; Aldeyab et al., 2008; Liebowitz and Blunt, 2008; Vernaz et al., 2008; Kaier et al., 2009; Jacoby et al., 2010; Thabet et al., 2010; Huang et al., 2011; Parienti et al., 2011; Bertrand et al., 2012; Lafaurie et al., 2012; Dancer et al., 2013; Couderc et al., 2014; Lawes et al., 2015). Nevertheless, a few researchers after controlling for multifold confounding factors failed to observe a significant association (Wibbenmeyer et al., 2010; Datta et al., 2014). In addition three papers reported that the use of distinct antibiotics from the group of fluoroquinolones had diverse effects on the rate for MRSA, contradicting partly to each other (MacDougall et al., 2005; Bosso and Mauldin, 2006; Salangsang et al., 2010). Moreover, as mentioned above, trends in the rate for MRSA and in the consumption of fluoroquinolone type antibiotics in Hungary during the past 15 years are also suggestive of a relationship (**Figures 1** and **2**). Despite this strong circumstantial evidence the abscence of an established mechanism for how fluoroquinolones could influence the rate of β-lactam resistance in *Staphylococcus aureus* might have precluded the acceptence of a causal relationship (Füzi, 2014).

An additional factor may complicate the situation somewhat further. One of the groups investigating a possible link between fluoroquinolone use and the rate for MRSA (Parienti et al., 2011) observed a decline in the incidence of MRSA subsequent both to a decrease and an increase in the consumption of the incriminated antibiotics. The mechanism described above posits that the development of resistance to fluoroquinolones is associated with a significant fitness cost in minor clone strains of MRSA (Horváth et al., 2012), however, the findings also imply that sooner or later all MRSA strains – even the most able isolates – will suffer some fitness cost when exposed to the pressure of fluoroquinolones in the long term, that may account for the observation of Parienti et al. (2011). Nevertheless, not surprisingly, Parienti et al. (2011) reported a much greater decline in the rate for MRSA if the use of fluoroquinolones was restricted relative to that associated with an increase in consumption. This “long-term fluoroquinolone pressure” on prevailing resident clones should also influence the rate for MRSA in the healthcare setting.

Since the recognition of novel lineages/sublineages of MRSA seem to be of utmost importance efficient techniques for the identification of newly emerging variants of the pathogen are warranted. Spa typing proved very useful in detecting variations within STs (Grundmann et al., 2014) and the continuous combined surveillance of spa types and multiple-locus variable number tandem repeat fingerprint (MLVF) types were reported to be an excellent means to monitor the dynamics of MRSA lineages and sublineages (Glasner et al., 2013). Nevertheless, it is the sequence-based approach that will provide a comprehensive and highly reliable account of lineage/sublineage distribution of the pathogen. Our results should help in establishing the disseminating potential of newly emerged lineages or sublineages. By determining the fluoroquinolone resistance-associated fitness cost of strains from newly emerged groups of MRSA (and various other pathogens) will allow the prediction of their “disseminating capacity” in ward where fluoroquinolones remain in extensive use.

The available literature on *C. difficile* is also relevant: the proportion of the “fluoroquinolone-related” clones is substantial (Tickler et al., 2014; Freeman et al., 2015); in addition many studies disclosed the clonal affiliation of the investigated isolates allowing for a specific monitoring of the “fluoroquinolone-related” ribotypes. Similarly, to MRSA an abundance of papers clearly shows that the incidence of *C. difficile* infections decreases subsequent to a reduction in the consumption of fluoroquinolone type antibiotics (Muto et al., 2007; Valiquette et al., 2007; Debast et al., 2009; Price et al., 2010; Wilcox et al., 2012; Dancer et al., 2013; Kanerva et al., 2015; Sarma et al., 2015; Gordon et al., 2016).

An additional strong argument for the influence of fluoroquinolone consumption on the clonal dynamics of *C. difficile* is the well-established fact that the incidence of the ribotype 027 strains remains significantly lower in pediatric units compared with adult ward (McFarland et al., 2016) which is in agreement with our finding with the major clones of ESBL-producing *K. pneumoniae* in Hungary (Szilágyi et al., 2010) and should reflect the differing use of fluoroquinolones in the two departments.

In contrast to MRSA and *C. difficile* the reliable impact of fluoroquinolone use on the incidence of ESBL-producing *K. pneumoniae* and ESBL-producing *E. coli* will be elucidated by future studies investigating the clonal distribution of the isolates.

The question arises how can the findings reviewed in this paper be reconciled with the abundant literature reporting only a slight fitness cost associated with antibiotic resistance and the observations that fitness cost suffered upon acquiring resistance can be reversed by developing compensatory mutations?

First, these studies cannot be compared with those reviewed here since none of them investigated a crucial aspect of the mechanism: the clonal affiliation of the isolates.

Second, fitness cost – or the abscence of it – associated with resistance to antimicrobial agents is often a function of the drug of choice. For instance resistance to streptomycin and rifampicin will often not result in any fitness cost or the suffered loss in vitality can readily be reversed by compensatory mutations (Björkman et al., 1998; Maisnier-Patin et al., 2002; Sander et al., 2002; Trindade et al., 2009; Brandis et al., 2015; Durão et al., 2015). In contrast resistance in fungi to amphotericin B (Vincent et al., 2013) and resistance in plasmodium to antimalarial agents (Rosenthal, 2013) are always associated with fitness cost that cannot be wholly recovered. As we have seen resistance to fluoroquinolones is usually associated with fitness cost, though it is a function of clonal affiliation and cannot be wholly recovered at higher MIC values (Horváth et al., 2012; Knight et al., 2012; Tóth et al., 2014).

Third, the fitness cost associated with resistance to antibiotics is often related to the level of resistance of the strain tested. Fluoroquinolone type antibiotics undoubtedly belong to this group. Several authors reported small fitness cost and sometimes slight fitness gain in strains with low level resistance to quinolones (Giraud et al., 2003; O’Regan et al., 2010; Baker et al., 2013; Fàbrega et al., 2014). However, fitness costs associated with higher MIC values were usually greater and could not be reversed (Giraud et al., 2003; Komp Lindgren et al., 2005; Marcusson et al., 2005; O’Regan et al., 2010; Pope et al., 2010; Horváth et al., 2012; Baker et al., 2013; Fàbrega et al., 2014; Tóth et al., 2014). Melnyk et al. (2015) recently reviewed much of the related literature and demonstrated that higher MIC values for many antibiotics are generally associated with higher fitness costs. They also observed that this trend unfortunately remains poorly explored since many of the investigators failed to disclose the MIC values of their isolates.

Moreover, the non-reversibility of fitness cost associated with resistance to fluoroquinolones in minor clone strains (Tóth et al., 2014) is strongly supported by international epidemiological data. The clonal landscape for the multiresistant pathogens mentioned above remains largely stable: usually a few big clones or STs are competing with each other for “territory.” If minor clone strains could easily reverse the fitness cost associated with resistance to fluorquinolones novel international clones of various multidrugresistant pathogens should regularly emerge and replace the “resident major clones,” something we have not been witnessing.

Though, the findings discussed above show an important role for fluoroquinolones in the selection and dissemination of multiresistant clones of various bacteria additional antibiotics should also have contributed to this process, thus, a more judicious use of antibiotics in general and of fluoroquinolones in particular could improve the antibiotic resistance situation.

The genetic base of the “fluoroquinolone selection mechanism” has in part already been elucidated. The data obtained to date clearly show that favorable mutations in the gyrase and topoisomerase IV genes play a crucial role in the development of high-level resistance to fluoroquinolones with a concurrent preservation of fitness. This has been demonstrated for *K. pneumoniae* (Tóth et al., 2014), *C. difficile* (Wasels et al., 2015) and a novel emerging lineage of the ST8 MRSA clone in America (Alam et al., 2015).

However, not all changes detected in the gyrase and topoisomerase genes proved favorable. We have observed a cluster of synonymous and non-synonymous mutations in the grlB gene of our ST30 MRSA strain that could have contributed to its compromised fitness relative to that of isolates from other clones of MRSA (Horváth et al., 2012).

Additional mechanisms associated with resistance to fluoroquinolones seem to command an inferior role compared with mutations in the gyrA and parC genes. In our *Klebsiella* study (Tóth et al., 2014) we failed to demonstrate the presence of the plasmid-mediated quinolone resistance determinants (PMQRDs): qnrA, qnrB, qnrC, qnrD, qnrS, qepA, and oqxAB in any of our isolates. In contrast all of the major clone strains and one of the minor clone isolates possessed the aac(6′)-lb-cr determinant that was related to the carriage of plasmids harboring CTX-M-15 ESBL genes. Moreover, none of the major clone strains but three of the four minor clone isolates showed efflux activity.

The data presented by us suggest that the ability of the reviewed bacteria to transmit between individuals is strongly influenced by the “speed of multiplication”; a trait we call fitness. This “speed of multiplication” can be reliably measured *in vitro* and seems to impact heavily on the disseminating capacity of the isolate. Data published on MRSA, ESBL-producing *K. pneumoniae* and *C. difficile* indicate that virulence factors play an inferior role in the dissemination of these species relative to fitness. A possible reason could be the competition of bacteria during the initial colonization stage. At this stage the “speed of multiplication” could often decide the “winner” and pathogenicity factors – certainly with the exception of those involved in attachment to host or killing other bacteria – remain less essential. If a strain is getting outgrown by another in the area of colonization it will have limited value of most of its pathogenicity factors which are directed against the host. This is, of course, not to deny that virulence factors of the reviewed pathogens could strongly impact dissemination when the fitness of the competing isolates remain equal. Nevertheless, fitness studies conducted with individual strains in animals *in vivo* remain less relevant for transmissibility than propagation assays performed *in vitro*, similarly, to competitive *in vivo* studies where the infection of the animal did not resemble the usual way of natural transmission.

However, in other species of bacteria virulence factors could assume greater significance in dissemination most certainly in a clone-affiliated fashion.

In conclusion we can say that:

(1) Major international clones of several multiresistant pathogens have emerged during the past three decades (see literature above).
(2) Experimental findings show that diverse fitness cost associated with resistance to fluoroquinolones could have influenced the clonal dynamics in MRSA, ESBL-producing *K. pneumoniae*, ESBL-producing *E. coli*, and multiresistant *C. difficile*. These findings are based on two lines of evidence.
  (a) The major international clones/lineages of MRSA, ESBL-producing *K. pneumoniae*, ESBL-producing *E. coli*, and *C. difficile* were shown to command favorable fitness when displaying high-level resistance to fluoroquinolones (Horváth et al., 2012; Knight et al., 2012; Tóth et al., 2014; Johnson et al., 2015; Wasels et al., 2015). For MRSA identical findings have been reported from two independent laboratories (Horváth et al., 2012; Knight et al., 2012).
  (b) Genetic investigations demonstrated that the ability to develop favorable mutations in the gyrase and topoisomerase IV genes constitutes a prerequisite for retaining fitness in MRSA, ESBL-producing *K. pneumoniae*, multiresistant *C. difficile*, and ESBL-producing *E. coli* when showing high-level resistance to fluoroquinolones (see literature above). Some additional mechanisms of resistance to fluoroquinolones in *K. pneumoniae* seem to be either expendable (qnr type resistance) or may not provide a viable alternative (efflux; Tóth et al., 2014). The same was reported on efflux in ESBL-producing *E. coli* (Marcusson et al., 2009; Johnson et al., 2015).
(3) *In vitro* observations concerning the fitness of various clones of MRSA (Horváth et al., 2012; Knight et al., 2012) are supported by a plethora of papers describing clonal shifts of the pathogen worlwide. All of the clonal replacements reported in the literature (see references above) are in agreement with the results of the fitness assays obtained by us (Horváth et al., 2012) and by Knight et al. (2012). Clonal shifts and clonal distributions observed with *C. difficile* also support the “fluoroquinolone mechanism” (Lee et al., 2014; McFarland et al., 2016).
(4) The observations that plasmids harboring the CTX-M-15 gene – in contrast to those with SHV type ESBL genes – proved impossible to eliminate from *K. pneumoniae* showing resistance to fluoroquinolones may account for the worldwide dissemination of this enzyme at the expense of SHV type ESBLs in this species (Tóth et al., 2014). The issue should be further investigated in both ESBL-producing *K. pneumoniae* and ESBL-producing *E. coli*.

The impact of fluoroquinolone consumption on the prevalence of MRSA and the major international clones of *C. difficile* is well-established (see literature above).

The influence of fluoroquinolone use on the incidence of ESBL-producing *K. pneumoniae* and ESBL-producing *E. coli* could be properly investigated by determining the clonal affiliation of the isolates and appreciating the change exclusively in the rates of those major clone strains which are known to have been selected by fluoroquinolones.

## Author Contributions

MF was head of the research team establishing the impact of fluoroquinolone resistance-associated fitness cost on the clonal dynamics of methicillin-resistant *Staphylococcus aureus* and ESBL-producing *Klebsiella pneumoniae*. He perused the literature related to clonality and fluoroquinolone resistance-associated fitness cost in various pathogens and compiled the manuscript himself. All the inferences presented in the paper have been drawn by him.

## Conflict of Interest Statement

The author declares that the research was conducted in the absence of any commercial or financial relationships that could be construed as a potential conflict of interest.

## Footnotes

1 http://ecdc.europa.eu/en/healthtopics/antimicrobial_resistance/database

2 http://ecdc.europa.eu/en/activities/surveillance/ESAC-Net/Pages/index.aspx

3 http://bigsdb.web.pasteur.fr/klebsiella/klebsiella.html

4 http://ecdc.europa.eu/en/healthtopics/antimicrobial_resistance/database

5 http://ecdc.europa.eu/en/activities/surveillance/ESAC-Net/Pages/index.aspx
